# Discovery and biological profile of pyridachlometyl

**DOI:** 10.1002/ps.8239

**Published:** 2024-06-09

**Authors:** Yuichi Matsuzaki, Makoto Kurahashi, Satoshi Watanabe, So Kiguchi, Toshiyuki Harada, Fukumatsu Iwahashi

**Affiliations:** ^1^ Health & Crop Sciences Research Laboratory Sumitomo Chemical Co. Takarazuka Japan

**Keywords:** pyridachlometyl, pyridazine, tubulin, fungicide resistance, bioisostere

## Abstract

Pyridachlometyl is a novel tubulin dynamics modulator fungicide developed by Sumitomo as a new agent designed to tackle fungicide resistance. Pyridachlometyl is being developed as a first‐in‐class molecule with an anti‐tubulin mode of action, the chemical structure of which is characterized by a unique tetrasubstituted pyridazine ring. The first commercial product ‘Fuseki flowable’ received initial registration in 2023 in Japan. The concepts of the discovery project, optimization of chemical structures, and biological profiles are reviewed herein. © 2024 The Author(s). *Pest Management Science* published by John Wiley & Sons Ltd on behalf of Society of Chemical Industry.

## Graphical abstract

Binding sites of pyridachlometyl and benzimidazole fungicide (carbendazim).

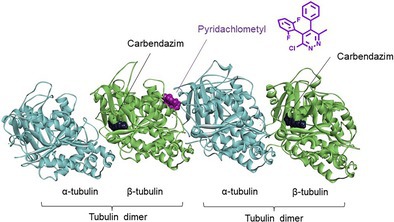

## 1 INTRODUCTION

Effective disease control is becoming progressively more difficult as fungal pathogens develop increasing levels of resistance to the major classes of fungicide.^1^, ^2^ The primary mechanism by which these fungi gain resistance is target‐site mutations, which impair the binding of fungicides to target proteins. Consequently, agrochemical companies and academic researchers are striving to identify novel modes of action based on the targeting of proteins that are not currently exploited as sites of fungicide action. However, broad‐spectrum fungicides tend to have similar mechanisms of action.^1^, ^2^ For example, multiple fungicides inhibit certain sites in the mitochondrial electron transport chain [quinone outside inhibitor (QoI), quinone inside inhibitor (QiI), and succinate dehydrogenase inhibitor (SDHI)], sterol biosynthesis [demethylation inhibitor (DMI) and amines], or tubulin polymerization (benzimidazole). Among the fungicides developed to date, benzimidazoles are tubulin‐inhibiting fungicides that target true fungi.^2^, ^3^ Contrastingly, in the field of medicine, different classes of tubulin inhibitor have been characterized that bind to seven distinct binding pockets in tubulin dimers comprising α‐ and β‐chains.^4^ These binding sites in tubulin dimers are discrete and spatially well separated. Consequently, the development of resistance against molecules targeting specific sites via target‐site mutations may not result in cross‐resistance against other molecules that bind to different pockets of tubulin dimers. Theoretically, if such molecules possess inhibitory activity against true fungi, they would be good candidates for a new class of agricultural fungicides. However, in this case, a possible hurdle to its application would be its potential toxicity to mammals, given that both mammals and true fungi are eukaryotic organisms belonging to the opisthokonts.^5^ Thereby highlighting the issue of selectivity.

## 2 START OF THE PROJECT

**Figure 1:**
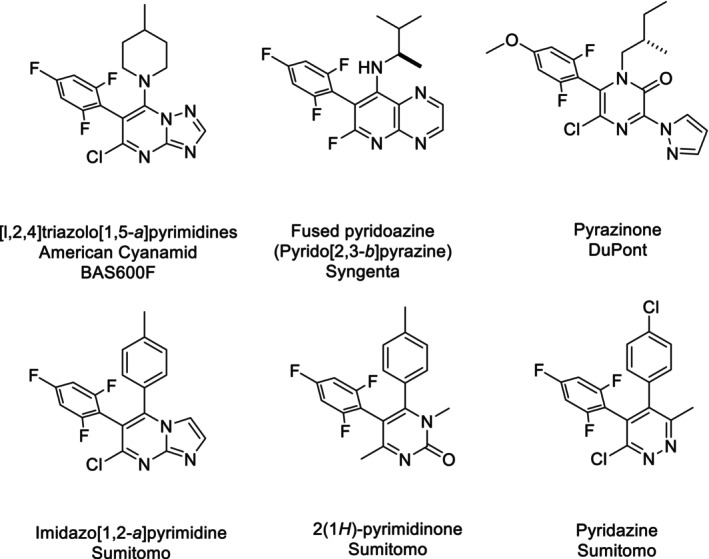
Chemical structures of experimental tubulin dynamics modulator fungicides.^6^, ^7^, ^8^, ^11^, ^12^, ^13^, ^15^

Toward the end of the 20th century, [l,2,4]triazolo[1,5‐*a*]pyrimidines (triazolopyrimidines), represented by BAS600F (Fig. 1),^6^ attracted the attention of researchers involved in fungicide discovery. As these compounds were characterized by pharmacophores that apparently differed from those of known fungicide groups, they were assumed to possess a novel mode of action. To the best of our knowledge, there are several research teams that have attempted to gain a proprietary lead based on bioisosteric transformation of the [l,2,4]triazolo[1,5‐*a*]pyrimidine pharmacophore. Among these, the Syngenta group was the first to report fused pyridoazine derivatives such as pyrido[2,3‐*b*]pyrazine,^7^ whereas researchers from DuPont have described pyrazinone derivatives.^8^ However, neither compounds with these heterocycles nor [l,2,4]triazolo[1,5‐*a*]pyrimidine pharmacophores have reached the stage of commercial development. In other studies, certain [l,2,4]triazolo[1,5‐*a*]pyrimidine analogs have been intensively investigated as anticancer medicines, owing to their high potency against mammalian tubulins and high bioavailability in mammals.^9^, ^10^ The findings of these studies indicate that the strong *in vitro* and *in vivo* effects of [l,2,4]triazolo[1,5‐*a*]pyrimidine on mammalian tubulins have important implications for the selectivity between fungi and mammals. Furthermore, compared with the activity observed in the laboratory, pyrido[2,3‐*b*]pyrazine has been reported to show poor efficacy under field conditions owing to the photodegradation of this heterocycle when exposed to sunlight.^7^

In addition to the aforementioned studies, Sumitomo's team has also examined the bioisosteric transformation of the [l,2,4]triazolo[1,5‐*a*]pyrimidine pharmacophore,^11^, ^12^ and found that the imidazo[1,2‐*a*]pyrimidine can function as a pharmacophore (Fig. 1).

## 3 BIOISOSTERE

**Figure 2:**
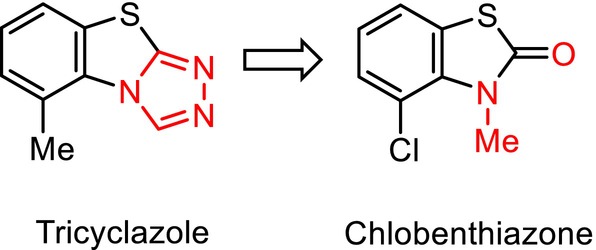
A related plausible bioisosteric replacement between amide and 1,2,4‐triazole moieties incorporated into or fused with heterocycles.^14^

**Figure 3:**
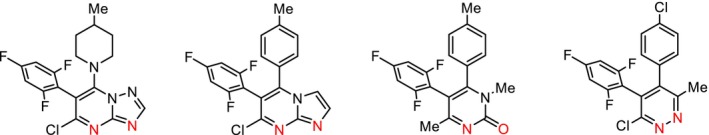
Hetero‐atoms (red color) of bicyclic and monocyclic pharmacophores as hypothetical hydrogen bond receptors at target sites.^11^

Although we identified imidazo[1,2‐*a*]pyrimidine as our proprietary lead compound, we speculated that bicyclic heterocycles with three different substituents could hinder high‐throughput synthesis and thereby limit the potential for structural simplification, which is advantageous for commercial manufacturing.^11^ Consequently, we assessed several bioisosteric monocyclic heterocycles as the pharmacophore.^11^, ^13^ In this context, previous studies on chlobenthiazone [4‐chloro‐3‐methylbenzothiazol‐2(3*H*)‐one], which is structurally derived from tricyclazole,^14^ have provided valuable clues indicating a potential bioisosteric relationship between the amide and 1,2,4‐triazole moieties when incorporated into or fused with heterocycles (Fig. 2). Based on the structure of imidazo[1,2‐*a*]pyrimidine, we designed and synthesized a 2(1*H*)‐pyrimidinone compound with a simplified structure (Figs 1 and 3). This 2(1*H*)‐pyrimidinone compound was found to be characterized by intrinsic broad‐spectrum fungicidal activity,^11^, ^13^ although its potency tended to vary depending on the target fungal species.^11^ Nonetheless, these promising results encouraged further examination of monocyclic heterocycles. It was assumed that the binding site of the target protein could accommodate different ligands and show sufficient flexibility to facilitate ligand interactions. Consequently, we designed and synthesized a pyridazine compound featuring two adjacent nitrogen atoms (Fig. 3).^11^, ^15^ The pyridazine compound shown in Fig. 1 was found to have more potent fungicidal activity than the 2(1*H*)‐pyrimidinone compound, with an efficacy comparable to that of the bicyclic heterocycles.^11^, ^15^, ^16^ Furthermore, the pyridazine compound showed sufficient photostability, which enabled long‐lasting control of diseases in fields exposed to sunlight.^17^

Interestingly, around the same time, a Merck research team in the pharmaceutical sector independently published a report acknowledging the structural similarity between 3,5‐disubstituted pyridazine and 3,7‐disubstituted imidazo[1,2‐*a*]pyrimidine as a benzodiazepine site‐selective agonist‐type anxiolytic agent.^18^

## 4 DISCOVERY OF PYRIDACHLOMETYL

**Figure 4:**
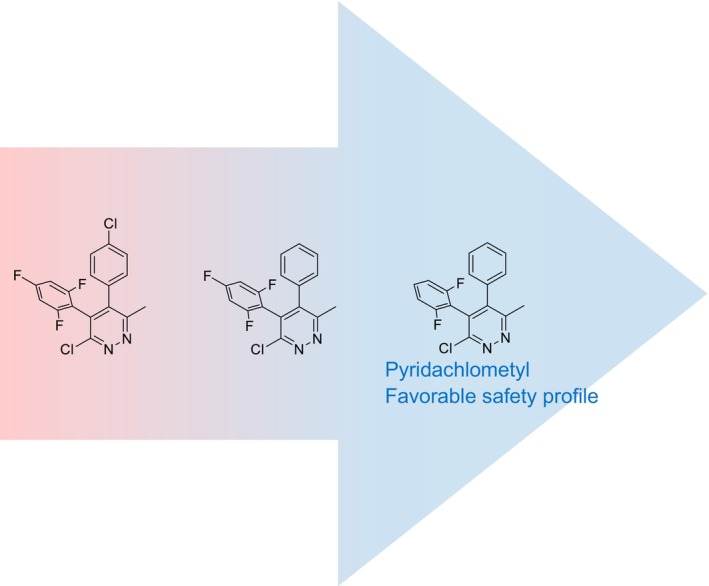
The selection of pyridachlometyl.^11^

Based on the structure–activity relationships of pyridazine compounds – published by the Syngenta group, who complemented our research efforts at Sumitomo Chemical^15^, ^16^ – we identified numerous analogs with high activity against different fungi. Among these pyridazine molecules, Sumitomo selected pyridachlometyl as a promising compound for further development, based on considerations of manufacturability and safety profile (Fig. 4, Tables 1, 2, 3, 4).^11^, ^19^

**Table 1:** Summary of the acute toxicity of pyridachlometyl^17^

Test type	Pyridachlometyl
Rat acute oral (LD_50_)	> 2000 mg kg^−1^
Rat acute dermal (LD_50_)	> 2000 mg kg^−1^
Rat inhalation (LC_50_)	> 5450 mg m^−3^ of air (4‐h, nose only exposure)
Eye irritation (Rabbit)	Minimal irritant
Skin irritation (Rabbit)	Minimal irritant
Skin sensitization (Guinea pig)	Sensitizer

*Note*: LC_50_, median lethal concentration; LD_50_, median lethal dose.

**Table 2:** Summary of the sub‐acute and chronic toxicity of pyridachlometyl^17^

Species	Administration route and duration	Dose (ppm)	NOAEL (mg kg d^−1^)
Dog	Oral (in capsules), 13 weeks	100, 300, and 1000 mg kg^−1^ d^−1^	Male: 100
			Female: 100
Dog	Oral (in capsules), 12 months	10, 50, and 300 mg kg^−1^ d^−1^	Male: 10
			Female: 10
Rat	Oral (in diet), 13 weeks	1000, 5000, and 20000	Male: 291 (5000 ppm)
			Female: 351 (5000 ppm)
Rat	Oral (in diet), 24 months	200, 2000, 10000, and 20000	Male: 8 (200 ppm)
			Female: 10 (200 ppm)
			No carcinogenicity
Mouse	Oral (in diet), 13 weeks	1500, 3500, and 7000	Male: 517 (3500 ppm)
			Female: 650 (3500 ppm)
Mouse	Oral (in diet), 18 months	700, 2000, and 7000	Male: 83 (700 ppm)
			Female: 317 (2000 ppm)
			No carcinogenicity

*Note*: NOAEL, no‐observed‐adverse‐effect‐level.

**Table 3:** Summary of the developmental and reproductive toxicity of pyridachlometyl^17^

Study	Species	Administration route and duration	Dose (mg kg^−1^ d^−1^)	NOAEL (mg kg^−1^ d^−1^)	
Developmental toxicity	Rat	Oral (gavage)	250, 500, and 1000	Maternal	1000
				Fetal	1000
		Days 6–19 of gestation			
	Rabbit	Oral (gavage)	250, 500, and 1000	Maternal	250
		Days 6–28 of gestation		Fetal	1000
Two‐generation reproductive toxicity	Rat	Oral (in diet)	600, 4000, and 20000 ppm	Parental	Male; 218 (4000 ppm)
					Female; 329 (4000 ppm)
				Offspring	Male; 267 (4000 ppm)
					Female; 362 (4000 ppm)
				Reproductive	Male; 1145 (20000 ppm)
					Female; 1697 (20000 ppm)

**Table 4:** Summary of the mutagenicity of pyridachlometyl^17^

Study	Study design	Results
Reverse mutation (Ames test)	*Salmonella typhimurium*: TA98, TA100, TA1535, and TA1537	Negative
	*Escherichia coli*: WP2uvrA	
	−/+S9 mix: 156–5000 μg per plate	
*In vitro* chromosomal aberration	Chinese hamster CHL/IU	Positive
	−S9 mix (6 h): 0.938–7.50 μg mL^−1^	
	+S9 mix (6 h): 30.0–50.0 μg mL^−1^	
Bone marrow micronucleus	CD‐1 mice	Negative
	500, 1000, and 2000 mg kg^−1^	

**Figure 5:**
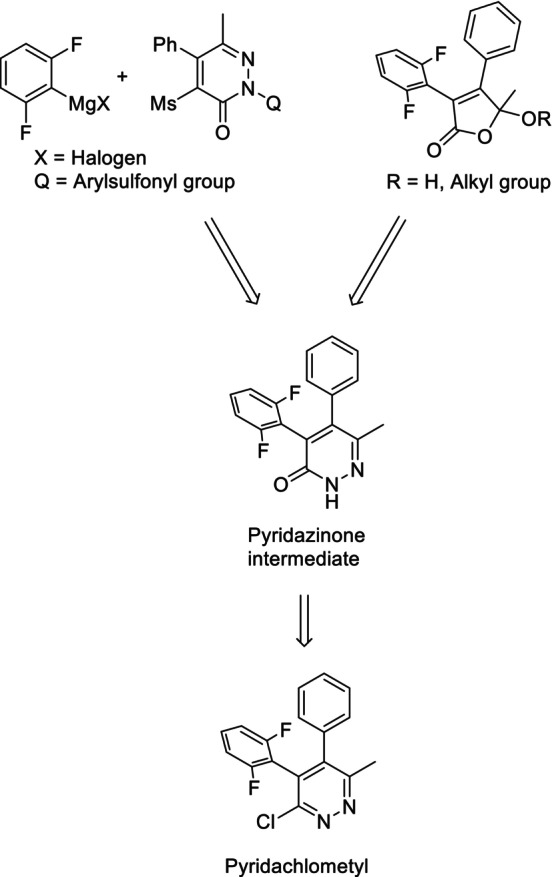
The route of pyridachlometyl synthesis.^11^, ^17^, ^19^, ^20^

In this regard, it has been established that the removal of para‐substituents from the two benzene rings facilitates reasonable manufacturing. Pyridachlometyl can be synthesized either by transforming a precursor with a 2,6‐difluorophenyl group via a pyridazinone intermediate or by directly introducing a 2,6‐difluorophenyl group into the pyridazinone structure via coupling with a Grignard reagent (Fig. 5).^17^, ^19^, ^20^

The favorable safety profile of pyridachlometyl can be attributed to its lower intrinsic activity against mammalian tubulins than [l,2,4]triazolo[1,5‐*a*]pyrimidine compounds, along with its rapid degradation in mammals.^17^, ^21^ The latter property not only ensure *in vivo* safety with respect to the anti‐tubulin effects but also to any secondary effects unrelated to the compound's biological mode of action as a fungicide.^17^, ^21^ In this regard, it was found that carbon‐14 (^14^C)‐labeled pyridachlometyl was rapidly metabolized and largely excreted in the feces when orally administered to rats.^17^, ^21^ When administered orally, the rate of absorption is estimated to be > 90%, with no residual or accumulative presence in tissues.^17^ The primary metabolic reactions of pyridachlometyl involve hydroxylation of the methyl group at the 6‐position of the pyridazine ring, followed by the formation of a carboxylic acid or glucuronic acid conjugation of the hydroxyl group, substitution of a chlorine with glutathione, and further metabolism to yield cysteine conjugates, thiol derivatives, or mercapturic acid conjugates.^17^ This contrasts favorably from the case of [l,2,4]triazolo[1,5‐*a*]pyrimidine derivatives, which tend to be resistant to metabolism as clinical medicines.^9^, ^10^

## 5 BIOLOGICAL ASPECTS

### 5.1 Mode of action

**Figure 6:**
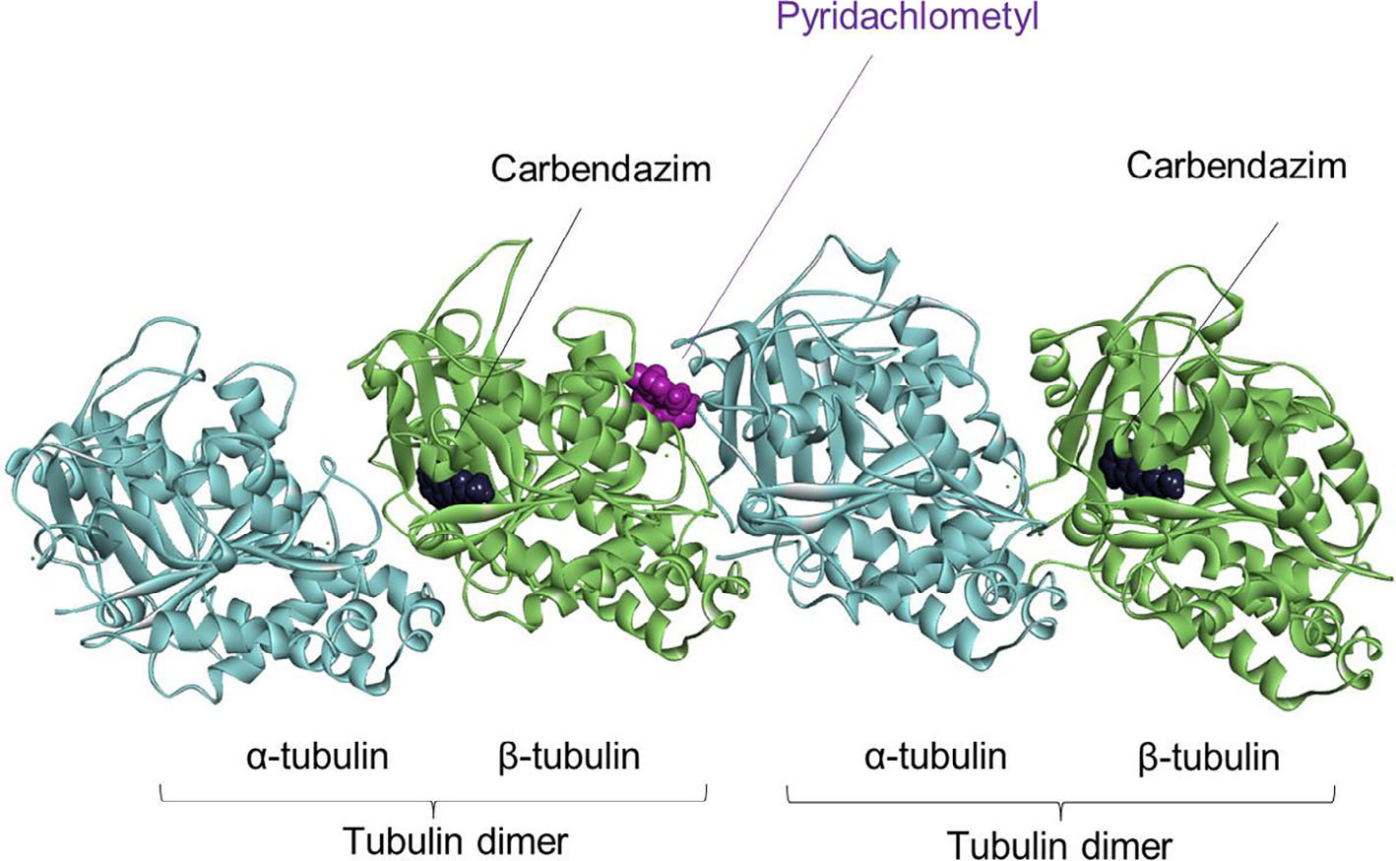
Binding sites of pyridachlometyl and the benzimidazole fungicide carbendazim (docking model^17^, ^19^). A homology model of *Zymoseptoria tritici* tubulin based on the X‐ray patterns of *Bos taurus*, *Sus barbatus*, and *Gallus gallus* (PDB, 5NJH, and 5C1A1) tubulins.

**Figure 7:**
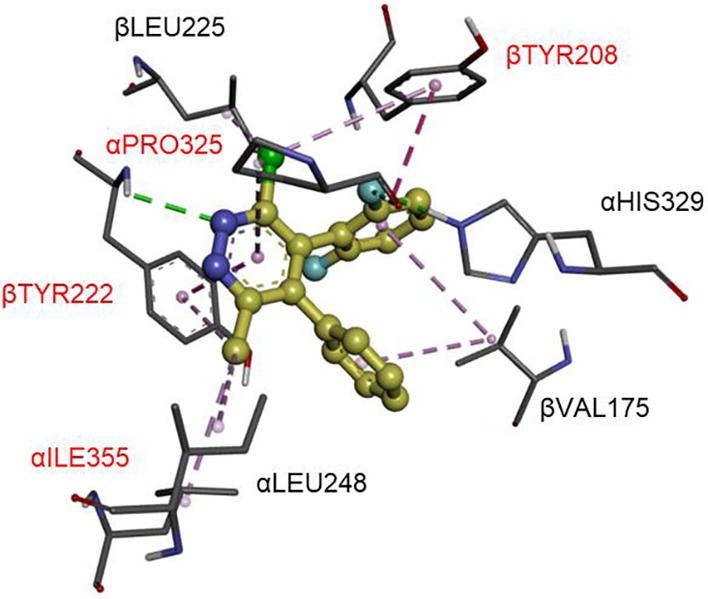
Close‐up view of the putative binding site of pyridachlometyl (docking model^17^, ^19^). A homology model of *Zymoseptoria tritici* tubulin based on the X‐ray patterns of *Bos taurus*, *Sus barbatus*, and *Gallus gallus* (PDB, 5NJH, and 5C1A1) tubulins. Altered amino acid residues resulting in a change in pyridachlomethyl sensitivity are shown in red.

The mode of action of pyridachlometyl and its analogs has been previously reported,^8^, ^16^, ^17^, ^22^, ^23^ on the basis of which, the molecule has been classified as a FRAC group 53 compound, distinct from the group 1 compounds that include benzimidazole fungicides such as carbendazim (https://www.frac.info/).^22^ Although pyridachlometyl targets tubulins, its binding site within tubulin dimer is a vinblastine‐binding site or a site in close proximity (Figs 6 and 7).^17^, ^22^ These findings were obtained in studies using pyridachlometyl‐resistant laboratory mutants and the homology model constructed based on the X‐ray structure of a triazolopyrimidine compound bound to a tubulin tetramer.^17^, ^22^, ^23^ Furthermore, unlike FRAC group 1 fungicides, pyridachlometyl is not classed as a ‘tubulin polymerization inhibitor,’ as *in vitro* studies have indicated that pyridazine molecules do not inhibit tubulin polymerization, but instead promote it.^16^ Among clinical tubulin‐targeting molecules, paclitaxel behaves similarly to pyridazines with respect to its polymerization or depolymerization activity, although its binding site is distinct.^10^ Thus, on the basis of findings regarding the mechanism of action of paclitaxel, it can be predicted that in living cells, pyridachlometyl would probably stabilize tubulin polymers and prevent their depolymerization.

### 5.2 Biokinetics

**Figure 8:**
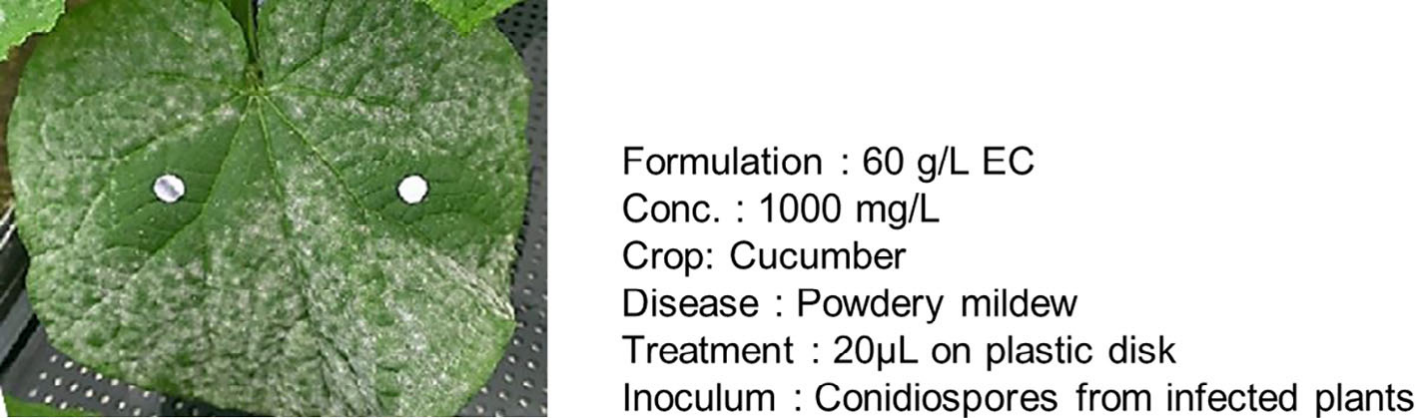
Circular zones of powdery mildew inhibition around pyridachlometyl‐impregnated plastic disks. Formulation, 60 g L^−1^ EC; Concentration of pyridachlometyl (active constituent), 1000 mg L^−1^; Treatment, 20 μL on plastic disk; Inoculum, conidiospores from infected plants.

In terms of biokinetics, redistribution via the gas phase is a notable property of pyridachlometyl. As shown in Fig. 8, clear circular zones of powdery mildew inhibition developed around the pyridachlometyl‐impregnated discs placed on mildew‐infected cucumber leaves. The circular shape indicates that redistribution does not occur via xylem flow, which only facilitates the movement of active constituents from the base (upstream) to the tip (downstream) of plant organs. In contrast, gas‐phase movement is not limited to unidirectional migration (Fig. 8).

**Figure 9:**
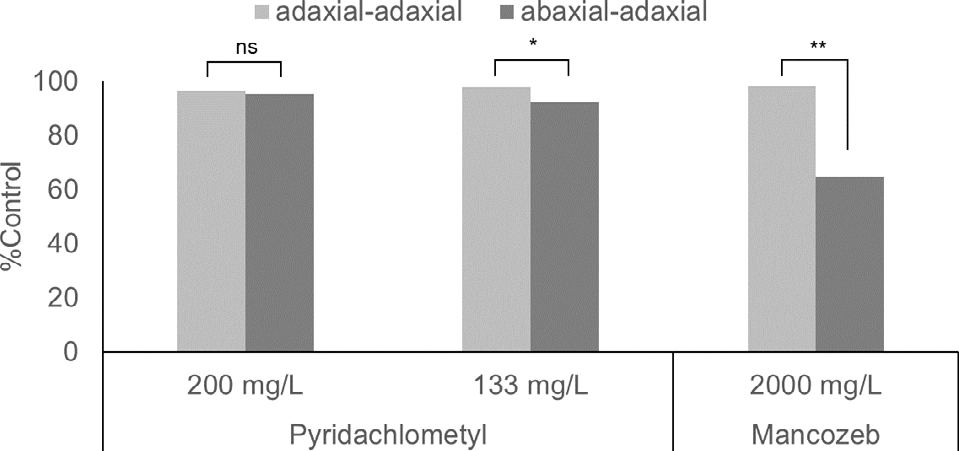
Translaminar activity of pyridachlometyl against cercospora leaf spot.^17^ Adaxial–adaxial: Test plants were inoculated with a conidial suspension of *Cercospora beticola* on the adaxial surface of the leaves 3 days after a fungicide had been applied to the adaxial surface. Abaxial–adaxial: Test plants were inoculated on the adaxial surface of leaves 3 days after the fungicide had been applied to the abaxial surface. Both tests were conducted in triplicate, with four leaves being assessed for each plant. Statistical differences were assessed using *t*‐tests. *P < 0.05, **P < 0.01.

Although systemic uptake from the root to foliar parts is estimated to be low, given that the logarithm of n‐octanol‐water partition coefficient (log*P*_ow_) is relatively high (4.10 at 20.5 ± 1.0 °C),^17^ pyridachlometyl exhibited a translaminar activity from the abaxial to adaxial surface of sugar beet leaves.^17^ The efficacy of pyridachlometyl on the adaxial surface of leaves when sprayed on the abaxial surface was sufficiently high compared with its efficacy on the abaxial surface, whereas the efficacy of mancozeb differed substantially (Fig. 9).^17^

### 5.3 Field performance

**Figure 10:**
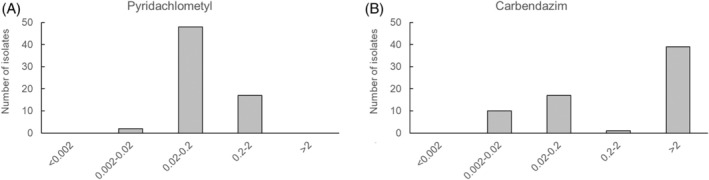
Histogram of half maximal effective concentration (EC_50_) values for pyridachlometyl (A) and carbendazim (B) in *Cercospora beticola* isolates.^17^ A total of 67 isolates collected from sugar beet fields in Hokkaido between 2014 and 2019 were assessed using a microtiter plate assay. EC_50_ values (mg L^−1^) were calculated for each fungicide.

**Figure 11:**
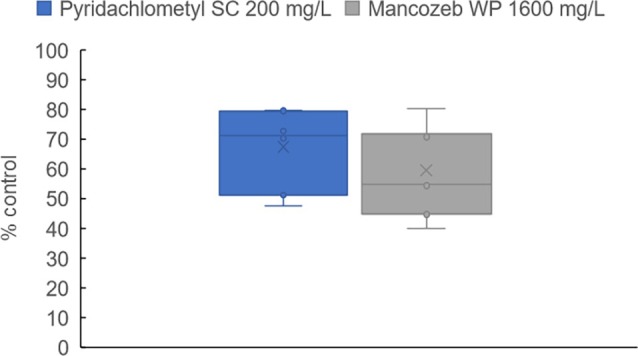
Results of field trials (sugar beet leaf spot caused by *Cercospora beticola*, Japan).^17^ In most trials, four to seven ground applications were performed using a water volume of approximately 1500 L ha^−1^ at intervals of approximately 7 days from the beginning of the primary infection.

A notable expectation with regards to pyridachlometyl is its inclusion as part of an anti‐resistance strategy in fungicide application programs, taking advantage of its novel mode of action. The distribution of the pyridachlometyl sensitivity of 67 *Cercospora beticola* isolates collected in Hokkaido, Japan, between 2014 and 2019 revealed a unimodal peak, whereas a bimodal peak was observed in the case of carbendazim (Fig. 10).^17^ In field trials, pyridachlometyl demonstrated a stable performance against *C. beticola* in Hokkaido, comparable to, or even surpassing, that of the multi‐site fungicide mancozeb at high concentrations (Fig. 11).^17^

**Figure 12:**
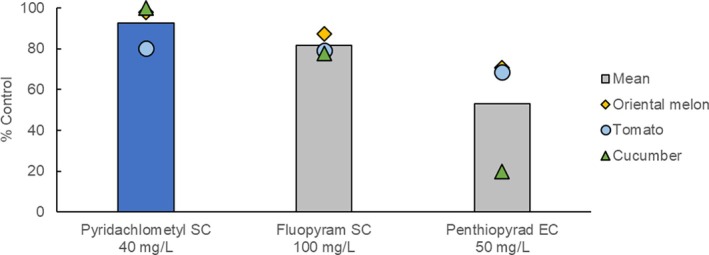
Results of field trials (vegetables/fruit, powdery mildews, Korea). For each crop, three ground applications were performed at a water volume of 1500 L ha^−1^ with a 7 days interval from the beginning of the primary infection.

**Figure 13:**
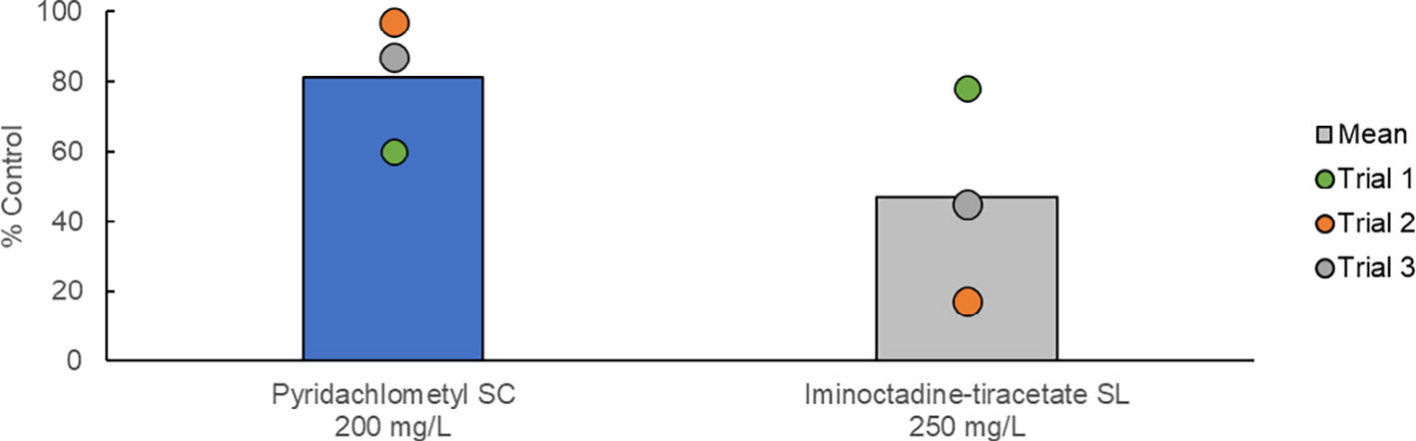
Results of field trials (wheat snow mold caused by *Microdochium* spp., Japan).^17^ A single ground application was performed prior to snow cover at water volumes of 1500–2500 L ha^−1^ at each site (*n* = 3).

In selected fruits and vegetables, pyridachlometyl was demonstrated to outperform the existing standard SDHIs in controlling powdery mildews (Fig. 12),^17^ whereas in wheat, pyridachlometyl was found to offer excellent protection from snow mold, *Microdochium* spp., which was superior to that obtained using the current standard, iminoctadine‐triacetate, which will lose pesticide registration in Japan in 2025 (Fig. 13).^17^ These findings indicated that pyridachlometyl is an ideal replacement.

**Figure 14:**
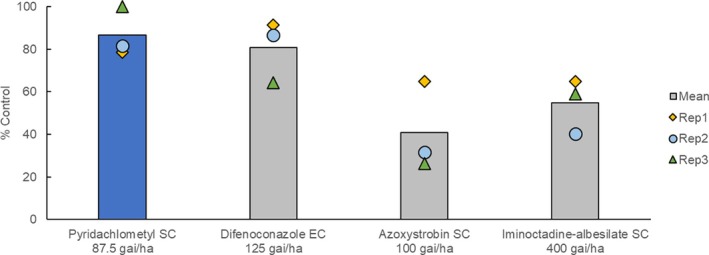
Result of a field trial using an unmanned aerial vehicle sprayer (soybean purple stain caused by *Cercospora kikuchii*, Japan). In a single trial with three replicate plots, a single application was performed using an unmanned aerial vehicle sprayer at 8 L ha^−1^ water volume after the flowering stage of soybean. The concentrations of the fungicides pyridachlometyl, difenoconazole, azoxystrobin, and iminoctadine‐albesilate in water were 1.09%, 1.56%, 1.25%, and 5%, respectively.

Moreover, in soybean, pyridachlometyl has been established to provide excellent control against *Cercospora kikuchii*, the causal agent of a serious end‐cycle disease, when applied using an unmanned aerial vehicle (UAV) as a sprayer (Fig. 14). Given the water volume restrictions associated with UAV application (< 10 L ha^−1^), this could result in insufficient coverage of spraying water on reproductive‐stage soybeans with ample foliage, thereby resulting in the non‐homogeneous adhesion of sprayed water.^24^ However, the redistribution of pyridachlometyl via the gaseous phase (Fig. 8) could potentially compensate for this deficiency, thereby contributing to the restoration of the uniformity of adhesion.

## 6 CONCLUSION

Pyridachlometyl is the first member of a new generation of tubulin‐targeting fungicides, defined as tubulin dynamics modulator. In addition to being effective, the reason behind selecting pyridachlometyl was its safety in terms of achieving a balance between chemical stability in both the field and mammalian body. In terms of practical applications, this compound is characterized by gas‐phase redistribution; as such, its usage is compatible with modern application technologies, such as UAVs.

Pyridachlometyl is a promising new fungicide currently under development, displaying excellent performance against a broad range of fungal pathogens. Our research has succeeded in providing a new product that will contribute to addressing the highly problematic resistance of fungal pathogens to currently applied fungicides.

## Data Availability

The data that support the findings of this study are available from the corresponding author upon reasonable request.
